# Development of a Hybrid-Imaging-Based Prognostic Index for Metastasized-Melanoma Patients in Whole-Body 18F-FDG PET/CT and PET/MRI Data

**DOI:** 10.3390/diagnostics12092102

**Published:** 2022-08-30

**Authors:** Thomas Küstner, Jonas Vogel, Tobias Hepp, Andrea Forschner, Christina Pfannenberg, Holger Schmidt, Nina F. Schwenzer, Konstantin Nikolaou, Christian la Fougère, Ferdinand Seith

**Affiliations:** 1 MIDAS.Lab, Department of Radiology, University Hospital of Tübingen, 72076 Tubingen, Germany; 2 Nuclear Medicine and Clinical Molecular Imaging, Department of Radiology, University Hospital Tübingen, 72076 Tubingen, Germany; 3 Department of Dermatology, University Hospital of Tübingen, 72070 Tubingen, Germany; 4 Department of Radiology, Diagnostic and Interventional Radiology, University Hospital of Tübingen, 72076 Tubingen, Germany; 5 Faculty of Medicine, Eberhard-Karls-University Tübingen, 72076 Tubingen, Germany; 6 Siemens Healthineers, 91052 Erlangen, Germany; 7 Cluster of Excellence iFIT (EXC 2180) Image-Guided and Functionally Instructed Tumor Therapies, Eberhard Karls University, 72076 Tubingen, Germany; 8 German Cancer Consortium (DKTK), German Cancer Research Center (DKFZ), Partner Site Tübingen, 72076 Tubingen, Germany

**Keywords:** melanoma, multiparametric PET/MRI, PET/CT, risk assessment, artificial intelligence

## Abstract

Besides tremendous treatment success in advanced melanoma patients, the rapid development of oncologic treatment options comes with increasingly high costs and can cause severe life-threatening side effects. For this purpose, predictive baseline biomarkers are becoming increasingly important for risk stratification and personalized treatment planning. Thus, the aim of this pilot study was the development of a prognostic tool for the risk stratification of the treatment response and mortality based on PET/MRI and PET/CT, including a convolutional neural network (CNN) for metastasized-melanoma patients before systemic-treatment initiation. The evaluation was based on 37 patients (19 f, 62 ± 13 y/o) with unresectable metastasized melanomas who underwent whole-body 18F-FDG PET/MRI and PET/CT scans on the same day before the initiation of therapy with checkpoint inhibitors and/or BRAF/MEK inhibitors. The overall survival (OS), therapy response, metastatically involved organs, number of lesions, total lesion glycolysis, total metabolic tumor volume (TMTV), peak standardized uptake value (SULpeak), diameter (Dmlesion) and mean apparent diffusion coefficient (ADCmean) were assessed. For each marker, a Kaplan–Meier analysis and the statistical significance (Wilcoxon test, paired *t*-test and Bonferroni correction) were assessed. Patients were divided into high- and low-risk groups depending on the OS and treatment response. The CNN segmentation and prediction utilized multimodality imaging data for a complementary in-depth risk analysis per patient. The following parameters correlated with longer OS: a TMTV < 50 mL; no metastases in the brain, bone, liver, spleen or pleura; ≤4 affected organ regions; no metastases; a Dmlesion > 37 mm or SULpeak < 1.3; a range of the ADCmean < 600 mm^2^/s. However, none of the parameters correlated significantly with the stratification of the patients into the high- or low-risk groups. For the CNN, the sensitivity, specificity, PPV and accuracy were 92%, 96%, 92% and 95%, respectively. Imaging biomarkers such as the metastatic involvement of specific organs, a high tumor burden, the presence of at least one large lesion or a high range of intermetastatic diffusivity were negative predictors for the OS, but the identification of high-risk patients was not feasible with the handcrafted parameters. In contrast, the proposed CNN supplied risk stratification with high specificity and sensitivity.

## 1. Introduction

Melanoma is the fifth leading cancer diagnosis [1], and the therapy for late-stage metastatic melanoma changed with the approval of checkpoint-inhibitor therapy (CIT), leading to a 5-year survival rate of more than 50% [2,3,4,5].

However, not all patients benefit from this therapy, and even with combined CIT (Ipilimumab + Nivolumab), only a 58% response rate can be achieved in the first-line setting [4]. The rate of treatment-related adverse events (59% Grade 3 and 4 events for Ipilimumab + Nivolumab [4]) and the treatment costs are high. Risk stratification before treatment initiation is therefore of great interest for personalized treatment decisions. Noninvasive imaging techniques enable the quantitative assessment of the individual tumor burden on the molecular level (by 8F-labeled fluorodeoxyglucose positron-emission tomography (18F-FDG-PET)), as well as on the morphological and functional levels (by multiparametric MRI), including diffusion-weighted imaging (DWI) [6]. For CIT, previous studies indicated a possibly predictive value of the metabolic activity of the primary and secondary lymphoid organs in 18F-FDG-PET [7,8,9]. It has been shown that intra- and intertumor heterogeneity has a tremendous impact on the clinical outcome and is of increasing interest for treatment planning [10,11,12]. In contrast to invasive biopsies, the imaging parameters can be assessed from the entire tumor burden, and thus enable a comprehensive overview of the patients’ tumor load that cannot be assessed by means of a biopsy. We hypothesize that there are imaging parameters for tumor aggressiveness that might correlate with a higher risk for a worse clinical outcome (i.e., shorter overall survival and treatment failure).

Oncologic imaging is moving towards quantitative-data extraction, and additionally, artificial-intelligence (AI) methods have been proposed to support the conventional reading [13] to gain deeper insights into the tumor biology [14], recently shifting from radiomic features towards data-driven deep-learning features [15]. The analysis of imaging data in oncology [16,17,18] mainly supports the automatization of the lesion segmentation [19,20,21], cancer classification (e.g., lung nodules in chest CT [22] or skin lesions [23,24]), disease classification [25,26,27], the detection of melanoma [28,29,30,31,32,33] and abnormalities and tumors [34,35,36,37,38]. Often, these models combine contextual and imaging information in an end-to-end fashion under the usage of multistream convolutional neural networks (CNNs) to accommodate multiple sources of information (e.g., imaging and nonimaging data) [39,40]. The usage of AI can help to further develop decision support systems that provide an objective evaluation of various treatment options. Moreover, it enables the development of radiomic-prediction models with better risk stratification for patients from a point-of-care perspective. The previously proposed approaches processed a variety of input data, ranging from diffusion-weighted MRI [41] over radiotherapy features [42], to photographic and dermoscopic images [30,43] to predict the overall survival (OS) for high-grade gliomas in MRI [44], the five-year disease-specific outcome in colorectal cancer from histology [45] or cervical cancer from PET/CT and MR examinations [46].

The aim of this pilot study was to evaluate the potential of different imaging markers based on multiparametric MRI, PET and CT to supply a risk stratification regarding the clinical outcome (OS and treatment response) of advanced melanoma patients undergoing systemic treatment. For this purpose, handcrafted parameters (metastatically involved organs, number of lesions, total metabolic tumor volume (TMTV), total lesion glycolysis (TLG), peak standardized uptake value (SULpeak), diameter (Dmlesion), mean apparent diffusion coefficient (ADCmean), spleen–liver ratio (SLR) and bone-marrow–liver ratio (BLR)) were extracted, and a multistream CNN was proposed. For the CNN analysis, the influence of the imaging modality and the correlation to the handcrafted parameters were investigated.

## 2. Material and Methods

**Figure 1:**
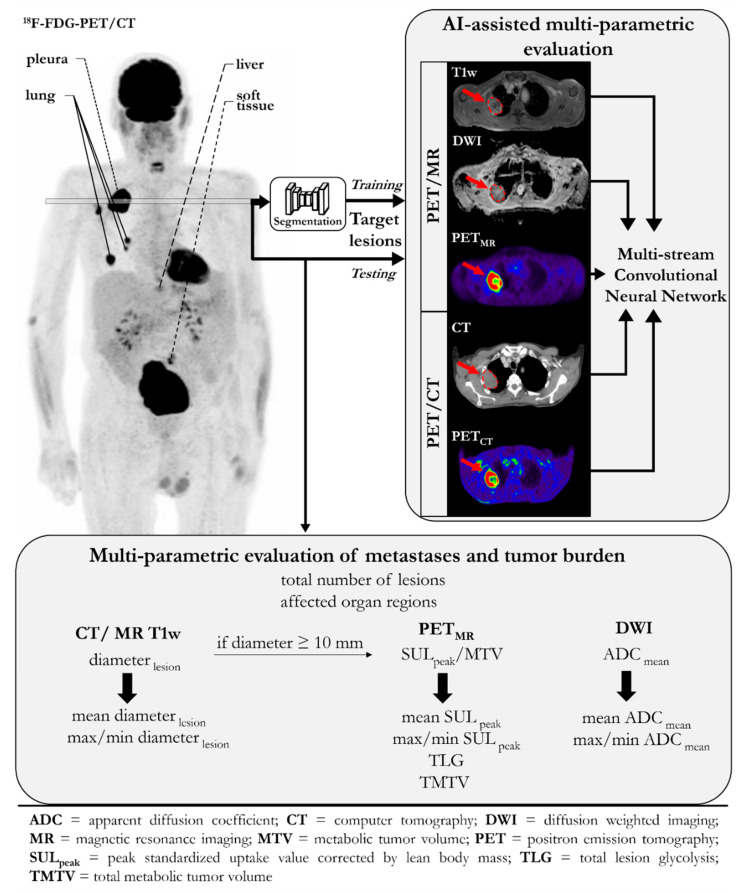
Overview of the multiparametric image analysis employing an AI-assisted evaluation and clinical evaluation to investigate the parametric dependency on treatment planning. An AI-assisted multiparametric evaluation was performed on automatically segmented (training) and manually selected (testing) target lesions (largest SULpeak and diameter) to support the treatment decision by grouping patients into low- and high-risk groups for unsuccessful immunotherapy treatment.

**Figure 2:**
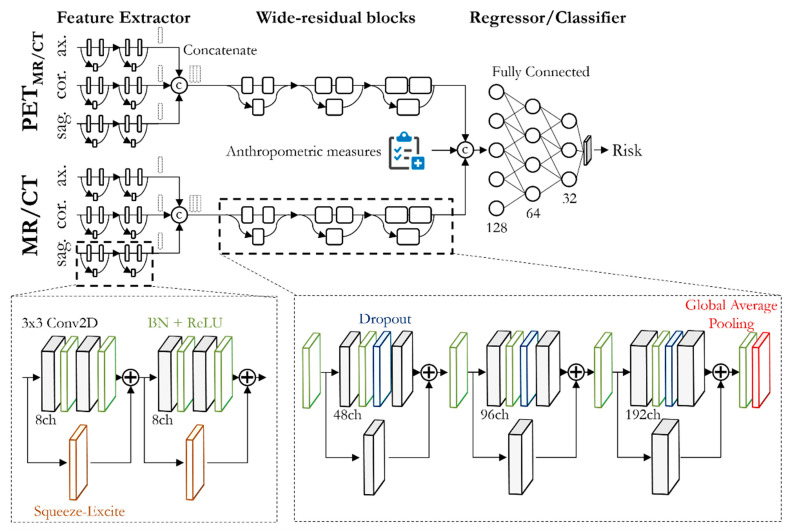
Proposed multistream CNN architecture for combined PET/MR/CT and MR/CT input to predict patient risk for failing immunotherapy treatment. Patients were considered low risk if the overall survival was >548 days and they showed a response to treatment. Each of the PET and MR/CT branches consists of three parallel 2D feature extractors for each image orientation (axial, coronal, sagittal) to enable a 3D processing of the input entity. A feature extractor is built up from two sequential squeeze-and-excite blocks consisting of two 3 × 3 2D convolutions with batch normalization (BN) and ReLU activation in parallel to a channel-wise excitation. Feature maps of each modality are processed in wide residual blocks, and they are then concatenated with anthropometric measures to form the desired prediction output in a fully connected classifier.

The study and analysis setup are depicted in Figure 1. Multiparametric multimodal PET/MR and PET/CT data were obtained in a prospective cohort study. Following the inclusion/exclusion criteria, 37 patients were selected for further analysis. A multiparametric evaluation of the metastasis and tumor burden was performed. A CNN (Figure 2) was trained on lesions in the cohort to assess the tumor aggressiveness (i.e., to classify patients into low-risk and high-risk groups that are categorized by the treatment response and overall survival). In order to reflect the clinical practice (i.e., only a smaller subset of lesions per patient was examined in detail to derive the treatment decision), the CNN was inferred with a single target lesion per patient.

### 2.1. Study Design

This prospective study was approved by the local ethics committee (code: 251/2012BO1) and the Federal Agency for Radiation Protection (code: Z5-22463/2-2012-023). It was registered on clinicaltrial.gov (NCT 03132090), as well as on the German clinical-trials register (DRKS00013925). Between September 2014 and March 2018, 62 patients were recruited. Written informed consent was obtained by all patients. Inclusion criteria for the study were as follows: (I) adult patients with (II) unresectable metastasized melanoma, (III) scheduled for systemic treatment with BRAF/MEK inhibitors, chemotherapy, anti-CTLA4/PD1 antibodies or a combination therapy. Exclusion criteria were as follows: acute infection of other acute diseases; pregnancy or breast feeding; disability for informed consent; clinical contraindications for MRI or a gadolinium-based contrast agent. Before treatment initiation, both whole-body 18F-FDG PET/CT and PET/MRI scans of each patient were performed. Previous studies have been published with this patient cohort to evaluate early changes in the metastases and lymphoid organs [7,47].

### 2.2. Patient Cohort and Outcome Evaluation

**Figure 3:**
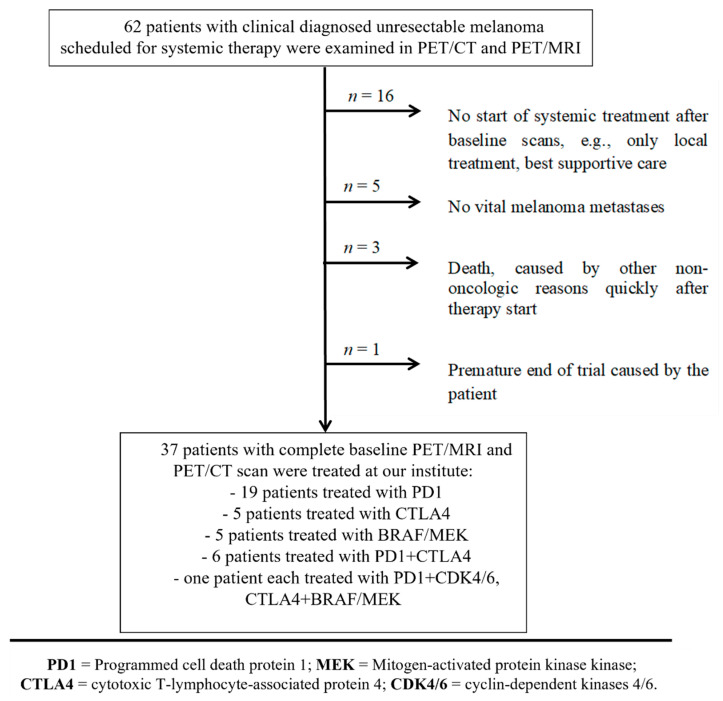
Flowchart for patient selection. From a cohort of 62 patients, 37 patients were selected based on the inclusion criteria.

For further evaluation, we included only patients with complete baseline 18F-FDG PET/CT and PET/MRI scans who were followed by systemic treatment at our institute. A flowchart for the patient selection is shown in Figure 3. An overview of the finally included 37 patients is shown in Table 1, resulting in a heterogeneous cohort, as is usually encountered in clinical routine. The OS was registered for all patients with a follow-up period of 1373 ± 372 days (at least 603 days), and this was last updated on 1 January 2020. The therapy response was evaluated three months after the start of therapy by whole-body 18F-FDG PET/CT (*n* = 28), or, in cases of obvious clinical progress, earlier by cross-section imaging (*n* = 9), and correlated to the decisions of the tumor board using RECIST 1.1 for patients treated with BRAF/MEK inhibitors, or iRECIST for patients treated with immunotherapy. In one case, where there was an inconsistency between the clinical decision (progress) and iRECIST (stable disease (tumor progression < 20%)), and the clinical decision was taken for further evaluation (Pat. 28, see Table 1).

### 2.3. PET/CT and PET/MRI Examination Parameters

At baseline (17 ± 16 days prior to treatment initiation), all patients underwent a clinically indicated 18F-FDG PET/CT scan (Biograph mCT; Siemens Healthcare GmbH, Erlangen, Germany), followed by a study examination in PET/MRI (Biograph mMR, Siemens Healthcare GmbH, Erlangen, Germany). Patients fasted for at least six hours prior to the injection of 18F-FDG (dose: 316 ± 13 MBq). The uptake time for the PET/CT was 60 min. The remaining activity was sufficient for the subsequent PET/MR imaging (uptake time: 120 min). In the PET/CT, a diagnostic contrast-enhanced CT (Ultravist 370; Bayer Healthcare) of the whole body (head to thighs, portal venous phase, expiratory breath-hold) was acquired. Additionally, a further scan of the thorax (inspiratory breath-hold) for the detection of lung metastases was performed. The CT acquisition and reconstruction parameters were as follows: 120–140 kV; reference dose: 200 mAs; pitch: 0.7; slice collimation: 128 × 0.6; gantry rotation time: 0.5 s; iterative reconstruction (Safire^®^, Siemens Healthcare GmbH, Erlangen, Germany) kernel B70 or I31f; slice thickness: 3 mm; increment: 2.5 mm; image resolution: 0.9 × 0.9 × 3 mm^3^; matrix size: 512 × 512 × 340—1000 (depending on the patient’s size).

For the PET/MRI, Gadobutrol (Gadovist (1.0 mmol/mL), Bayer Vital GmbH, Leverkusen, Germany) was used as a contrast agent. For the image evaluation, an axial whole-body fat-saturated post-contrast 3D T1-weighted volumetric interpolated breath-hold examination (VIBE) was acquired with the following parameters: TE/TR: 1.26/3.97 ms; flip angle: 12°; bandwidth: 440 Hz/px; resolution: 1.7 × 1.7 × 3 mm^3^; matrix size: 320 × 210 × 320; 448 multiple consecutive breath-holds. For the DWI, a free-breathing 3D whole-body axial DWI sequence with the following parameters was acquired: TE/TR: 0.9/2.63 ms; flip angle: 7°; bandwidth: 340 Hz/px; resolution: 1.8 × 1.8 × 3 mm^3^; matrix size: 256 × 256 × 320; no. of averages: 3; b-values: 50 and 800 s/mm2. ADC maps were calculated by the vendor’s software.

Whole-body PET scans were performed with a usual scan time per bed position of 2 min in PET/CT, and 4 min in PET/MRI. The PET reconstruction parameters of the PET/CT were as follows: 3D OP-OSEM time-of-flight reconstruction; 2 mm Gaussian filter; matrix size: 400 × 400. For the attenuation correction, the whole-body CT scan was used. The PET reconstruction parameters of the PET/MR were as follows: 3D ordered subset expectation maximization algorithm (3D OSEM); 21 subsets; 2 iterations; 4 mm Gaussian filter; matrix size: 256 × 256. For the attenuation correction, a 3D T1-weighted spoiled dual gradient-echo sequence with Dixon-based fat–water separation in end-expiratory breath-hold was used. The attenuation maps were carefully checked for erroneous tissue identification.

### 2.4. Multiparametric Evaluation of Metastases and Tumor Burden

Image analysis was carried out using Syngo.via software (Siemens Healthcare GmbH, Erlangen, Germany), in consensus with a senior radiologist (SF) with 7 years of experience in hybrid imaging, and a nuclear medicine physician (VJ) with 3 years of experience. For the PET evaluations, the PET/MR was taken due to the perfect alignment to MRI. We identified all metastases of a patient, and the number ($n_{lesion}$) was registered to a maximum of $n_{lesion}$ = 200. If n>200 lesions were found, then the number was set to $n_{lesion}$ = 200. A metastatic involvement was recorded in the following organ regions: lymph nodes, soft tissue, bone, liver, spleen, lung, pleura, brain and other viscera. Diameters of lesions were measured in the postcontrast VIBE sequence in MRI, except for lung metastases; lung metastases were evaluated in PET/CT, as CT is superior to MRI in the detection of small lung lesions [48]. In lesions with a longest diameter > 10 mm, the longest diameter (Dmlesion) in the axial plane, the peak standardized uptake value normalized by the lean-body-mass SULpeak and the ADCmean of a lesion were measured. The ADCmean was measured by drawing a free-hand ROI in the region of the largest diameter of the lesion, avoiding organ borders. If the region of a metastasis was severely affected by artifacts in the ADC map, then the ADCmean was not acquired. For brain metastasis, only the Dmlesion was measured. In lesions with a longest diameter < 10 mm, the diameter was set to 5 mm (assuming a diameter range between >0 and 10 mm), and no functional parameters were measured to avoid unprecise measurements caused by partial-volume effects. Mean, minimum and maximum values were calculated for the Dmlesion, SULpeak and ADCmean in each patient. Moreover, the TMTV and TLG were assessed by summing up the individual MTV and TLG values of each lesion of a patient using the PET/MR dataset. The MTV was defined as the volume enclosed by a 42% isocontour around the maximum PET voxel of tumor lesions, as described previously [49]. The total lesion glycolysis (TLG) was obtained by multiplying the MTV of each focal lesion with the mean SUL in the MTV. For the CNN evaluation (as described in the following section), a target lesion was defined and manually segmented in both PET/CT and PET/MRI. The target lesion was chosen as the lesion with the highest tracer uptake (independent on the lesion location) under the hypothesis of it being a representative surrogate for the tumor burden of the patient. Usually, this lesion was also the largest lesion of a patient. If there were several lesions with similar SULpeak and different diameters, then the largest lesion was chosen as the target lesion. Brain lesions were not included in this evaluation.

The SLR and BLR in PET were described as possibly predictive for the treatment outcome in CIT in the literature [7,8]. We performed an analysis in the subgroup of patients treated with CIT (*n* = 32) following Wong et al., and we set a VOI with a diameter of 2 cm in the liver and spleen to measure the SUL and compute a ratio of the SULmean spleen/SULmean liver [8]. The cut-off value was first chosen as 1.1, as described by Wong et al., and afterwards, the stepwise decreased down to 0.9 (step size: 0.05). According to Seban et al., SUL bone marrow is distinguished by averaging the SUL out of up to four VOIs (Dm = 1.5 cm), placed in tumor-free vertebrae L1–L4, and then related to the SUL liver. Vertebrae with severe lumbar osteoarthritis, vertebral fractures, hemangiomas or a history of lumbar spine surgery were excluded [50].

### 2.5. Convolutional Neural Network

A multistream CNN is proposed that investigates the tumor aggressiveness on the hybrid imaging data of the initial staging. Because tumor aggressiveness cannot be quantified and measured, the overall survival (OS) and treatment response acted as surrogate markers to identify low-risk and high-risk patients. Low-risk patients were characterized by an OS > 548 days and response to treatment; otherwise, patients were assigned to the high-risk group (i.e., OS ≤ 548 days or nonresponders, as indicated in Table 1). The cutoff was chosen according to Larkin et al., showing a minimum median OS of 19.9 months for patients with advanced melanoma undergoing treatment [4]. Cropped lesions of the coregistered PET/CT and CT images, as well as of the coregistered PET/MR and MR (postcontrast T1-weighted VIBE and DWI) images were fed into the network, together with the anthropometric measures (patient height, patient weight and lesion diameter).

The proposed architecture is depicted in Figure 2. The network has two main branches: a PET_MR/CT_ branch and an MR/CT branch, which, in each case, consist of three parallel feature extractors for each image orientation (axial, coronal, sagittal). This enables the use of 2D convolutions, which reduces the number of trainable parameters while still providing 3D processing. The feature extractor is built up from two sequential squeeze-and-excitation blocks, with two 3 × 3 2D convolutions (8 channels), batch normalization and the ReLU activation function in parallel to a 1 × 1 channel attention (consisting of global average pooling, fully connected, ReLU activation, fully connected, and sigmoid activation). Afterwards, orientational feature maps are concatenated and fed to a common stage of wide residual blocks per modality. The wide residual blocks have an increasing size of channels per block to cope with feature reusage [51]. Each wide residual block consists of a 3 × 3 2D convolution, batch normalization, ReLU activation, dropout, 3 × 3 2D convolution and a 3 × 3 2D convolution in the residual path. The feature maps are merged and subsequently processed through batch normalization and ReLU activation. At the end of the three wide residual blocks, a global average pooling is performed. The pooled feature maps of the PET_MR/CT_ and MR/CT branches are then merged with the anthropometric measures to be fed into a fully connected network with three dense layers and softmax activation for binary risk classification.

The PET_CT_ and CT images, as well as PET_MR_ and MR images, were first rigidly coregistered. The registration accuracy was verified manually. A separate segmentation network trained on PET/CT images of melanoma and bronchial-carcinoma patients [52] was applied to segment lesions. The lesion fields of view around the segmented lesions were taken with a common size of 132 × 160 × 250 mm (LR × AP × HF), which was determined by the largest lesion in the cohort. The segmented lesion with the highest uptake per patient was considered for training. DWI data were excluded if they were affected by artefacts in this region. The lesion diameter was automatically derived from the segmentation and fed into the network as an anthropometric feature. From these lesion fields of view, patches of a size of 32 × 32 × 32 (LR × AP × HF) were extracted. Slicing along the different patch dimensions provided 32 2D patches of the respective axial, coronal and sagittal input orientations. Data pairs of PET_MR_ + MR_VIBE_, PET_MR_ + MR_DWI_ and PET_CT_ + CT were used jointly as the network input.

The network was trained end to end using an Adam optimizer (learning rate: 0.0005; batch size: 32) over 150 epochs on a Nvidia Tesla V-100 GPU with categorical cross-entropy loss for risk assessment. Overall, the network resulted in ~1.8 million trainable parameters.

To eliminate patient bias, a 37-fold patient leave-out cross-validation was performed, (i.e., 37 trainings were performed each on 36 patients, and 1 patient left out for testing). If not stated otherwise, all results are reported as the average overall cross-validation runs. For the training, an empirically optimized number of 384 patches per patient was taken at random locations within the lesion field of view, yielding a training set of ~1.32 million 2D patches (for 36 subjects). For the validation, the lesion of the segmentation network with the second highest SULpeak and a minimum longest diameter (Dmlesion) of 10 mm per patient was automatically selected as the validation set, yielding a total of ~177.000 2D patches (for 36 patients). For the testing, the expert-labeled target lesion of the left-out subject (i.e., subject not seen in training) was selected, yielding ~3.800 2D patches per patient, with a 37-fold cross-validation.

Further experiments were conducted to investigate the influence of the imaging modality on the risk stratification. The training and testing performances were evaluated for the individual imaging modalities (PET_CT_ + CT or PET_MR_ + MR) in comparison with the proposed joint training on PET_MR_ + MR (PET_MR_ + MR_VIBE_, PET_MR_ + MR_DWI_) and PET_CT_ + CT. The performance of the proposed multistream CNN was compared against the labeled ground truth in terms of:

(1) $$\mathrm{specificity}\;=\frac{\mathrm{TN}}{\mathrm{TN}+\mathrm{FP}}$$

(2) $$\mathrm{sensitivity}\;=\frac{\mathrm{TP}}{\mathrm{TP}+\mathrm{FN}}$$

(3) $$\mathrm{positive}\;\mathrm{predictive}\;\mathrm{value}\;=\;\mathrm{PPV}\;=\frac{\mathrm{TP}}{\mathrm{TP}+\mathrm{FP}}$$

(4) $$\mathrm{accuracy}\;=\frac{\mathrm{TP}+\mathrm{TN}}{\mathrm{TP}+\mathrm{TN}+\mathrm{FP}+\mathrm{FN}}$$

which are derived from the confusion matrix between the predicted risk and the labeled ground truth. TP describes the true-positive samples of being low risk, TN the true-negative samples of being high risk, FP the false-positive samples and FN the false-negative samples.

### 2.6. Statistics

For the survival analysis, the patients were divided into two groups, and Kaplan–Meier analyses were performed. If nondichotomous variables were used, then hierarchic clustering was employed to maximize the dissimilarity between the two groups, with each group containing at least 10 patients. A Wilcoxon test was used to evaluate the differences in the OS between the two groups. For the treatment-response analysis, a *t*-test under the null hypothesis of equal means for unequal variances was performed. Multivariate analysis using the Cox-regression model was not feasible due to the small number of patients included in this study [53]. To counteract the problem of multiple comparisons, the Holm Bonferroni method was used to correct the local alpha level. The significance level of the *p*-values was 0.05. All the statistical analyses were performed using JMP^®^ (Version 13.1, SAS Institute Corporation, Heidelberg, Germany). The values are expressed as mean values ± one standard deviation or mean values (range).

## 3. Results

### 3.1. Treatment Outcome and Overall Survival

Out of the 37 patients evaluated in this study, 21 died during the follow-up period, and the median OS was 429 (120–1266) days. A total of 14 patients were classified as responders (complete response: *n* = 4; partial response: *n* = 8; stable disease: *n* = 2), and 23 patients were classified as nonresponders (progressive disease: *n* = 23). There was no impact of age or sex on the OS or treatment response.

### 3.2. Multiparametric Evaluation of Metastases and Tumor Burden

**Figure 4:**
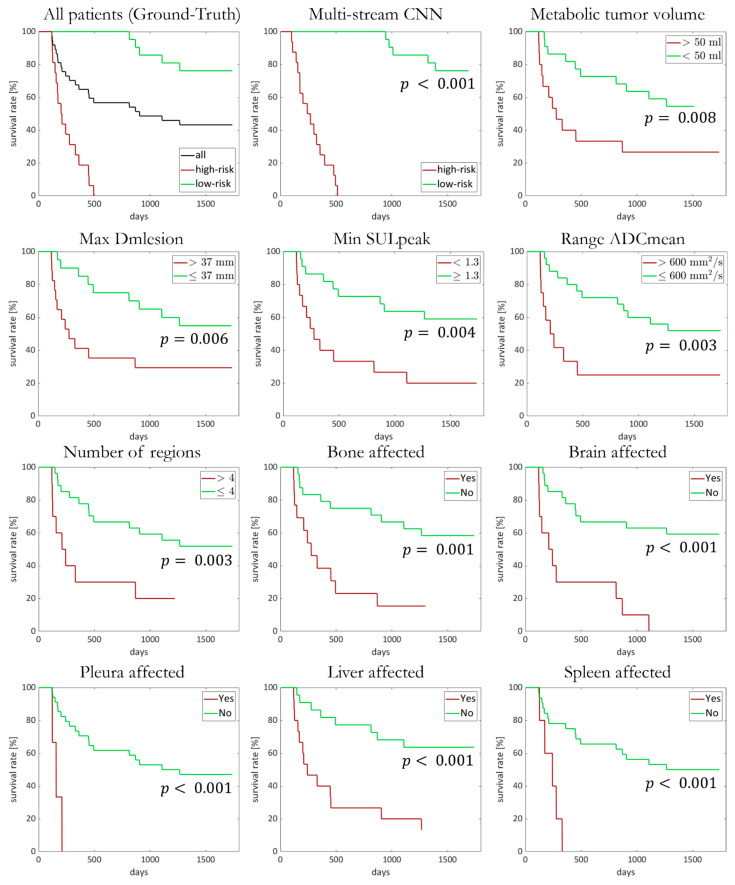
Kaplan–Meier plot for overall-survival analysis since start of systemic treatment for all patients depending on metabolic tumor volume, max. lesion diameter (Dmlesion), min. peak standardized uptake value (SULpeak), range of mean apparent diffusion coefficient (ADCmean), number of affected regions and respective affected regions, as well as estimated values obtained from multistream CNN.

In our cohort, a total of 972 lesions were found and evaluated further. An overview of the results is given in Table 2. All the Kaplan–Meier plots with significant differences between the groups are shown in Figure 4 and Figure S1.

Patients suffering from a TMTV ≤ 50 mL or ≤4 affected organ regions showed a significantly longer OS (*p* = 0.008 and *p* = 0.003, respectively). For the total number of lesions ($n_{lesion}$) and TLG, there was a trend for lower magnitudes to correlate with a longer OS, but this was not significant (*p* > 0.05).

The OS was significantly shorter if there were metastases present in the brain, pleura, liver, spleen or bone (*p* < 0.001). The affection of one of the other organ regions had no effect on the OS.

In addition, the OS was significantly affected in patients presenting with at least one lesion with a high Dmlesion (max. Dmlesion ≥ 37 mm; median OS: 277 vs. 1266 days; *p* = 0.006), a high intraindividual range of the ADCmean (range ADCmean ≥ 600 mm^2^/s; median OS: 210 days vs. range ADCmean ≤ 600 mm^2^/s and 1266 days; *p* = 0.003). Furthermore, the presence of at least one lesion with an SULpeak value under 1.3 was leading to a significantly shorter OS (*p* = 0.004).

An increased SLR > 1.1, described as as bad prognostic factor for OS [8], could only be measured in one patient. The OS of this patient was shorter than the median OS, but not substantial (243 days vs. 1266 days). By stepwise decreasing the cut-off value down to 0.9 (step size: 0.05), the number of patients fulfilling the requirement could be increased, but there was no cut-off value that resulted in a significant difference between both groups regarding the OS. The mean BLR was 0.76 ± 0.19. There was no effect of the BLR on the OS.

There was no significant impact on the treatment response (responders vs. nonresponders) of any of the evaluated parameters. Regarding the affected organ regions, there was a trend for a therapy response if there was no affection of the brain, bone, liver or pleura, but this was not significant anyway.

### 3.3. Convolutional Neural Network

The CNN prediction for primary target lesions utilizing both imaging modalities showed a 96% specificity, 92% sensitivity, 92% PPV and 95% accuracy to determine the risk of a patient for short survival or not responding to therapy within the ~8 s processing time over all cross-validations.

The contribution of each imaging modality to the overall performance was investigated. For the network trained on both imaging modalities, we observed an overall statistically significant higher sensitivity and precision for PET_CT_ + CT compared with PET_MR_ + MR, resulting in an 86%/88% specificity, 83%/35% sensitivity, 86%/61% PPV and 84%/71% accuracy if tested on the primary target lesion (*p* < 0.001). Hence, to minimize the potential bias of the trained network towards either one of the imaging modalities, the network was retrained and tested on a single imaging modality. For the training and testing on the target lesions in the PET/CT data, a 92% specificity, 93% sensitivity, 85% PPV and 92% accuracy were obtained. For the training and testing on the target lesions in the PET/MR, a 96% specificity, 38% sensitivity, 83% PPV and 77% accuracy were achieved. PET/MR is again statistically significantly (*p* < 0.001) less sensitive and statistically significantly (*p* < 0.001) less accurate than PET/CT. However, PET/MR yields a higher specificity, which can be attributed to the fact that the anatomical (VIBE) imaging data were paired with quantitative ADC maps (DWI).

The Kaplan–Meier plot in Figure 4 shows the OS for each of the respective predicted groups (network trained and tested on both imaging modalities). The patients in the high-risk groups survived no longer than 503 days. At the end of the observation period (1800 days), 76% of the low-risk patients were alive.

**Figure 5:**
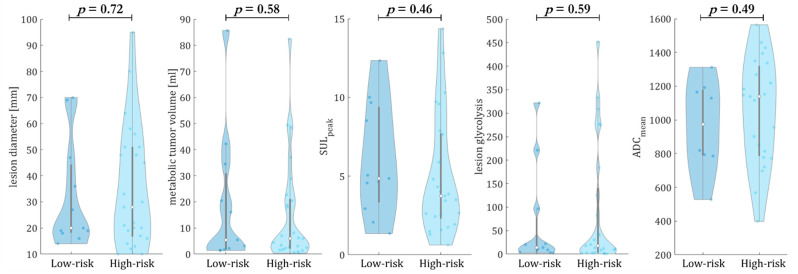
Analysis of CNN-based risk stratification in relation to clinical extracted parameters of the primary target lesions: lesion diameter, metabolic tumor volume, maximal standardized uptake value normalized by lean body mass (SULpeak), primary target lesion glycolysis and apparent diffusion coefficient (ADCmean). The CNN estimated the risk of the patients for treatment failure. No clinical parameter correlated statistically significantly with the predicted groupings into low- and high-risk groups obtained from the CNN, from which we concluded that the imaging-derived features and the lesions’ textural composition primarily determined the prediction.

In Figure 5, the risk-prediction analysis tested on both imaging modalities is set in relation to the prognostic multiparametric clinical parameters. No statistically significant parameter was observed (*p* > 0.05) to group patients into low- and high-risk groups. We therefore hypothesized that the network decided from the imaging features, and particularly from the lesions’ textural composition and its surrounding environment.

**Figure 6:**
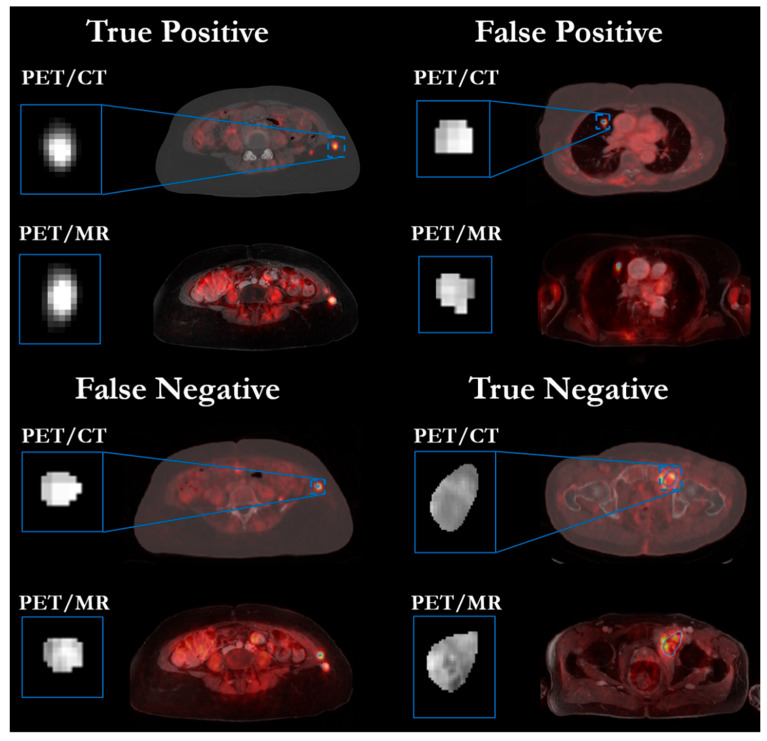
PET/MR and PET/CT images of four patients based on their CNN risk-classification output towards a true positive (true low risk), false positive (predicted low risk, but high-risk target), false negative (predicted high risk, but low-risk target) and true negative (true high risk). The PET images are color-coded overlaid to the CT/MR images. A zoomed version of the PET lesion is depicted to appreciate the textural inhomogeneity. The textural composition of the lesion guided the risk prediction.

To examine this assumption further, the visual representation in Figure 6 shows true-positive, false-positive, false-negative and true-negative cases of being low- or high-risk regarding the survival analysis. PET/CT and PET/MR images are shown, as well as the manual delineated lesion in the PET images. Some textural differences can be observed in the respective lesions for the true positive and true negative, from which we concluded that the multistream CNN had learnt to identify these structural inhomogeneities. Furthermore, misclassifications occurred mostly in small lesions close to a 1 cm diameter.

## 4. Discussion

The results of our pilot study confirm that certain imaging biomarkers, such as a high tumor burden or the metastatic involvement of specific organs, such as the brain, pleura, liver, spleen or bone, correlate with the shorter OS of patients. Moreover, the presence of metastases with large diameters, low metabolism (i.e., SULpeak) or a high intraindividual range of diffusivity (i.e., ADCmean) between all metastases of a patient also showed a significant and negative influence on the long-term clinical outcome. We demonstrated a multistream CNN that extracts image data from PET, MRI and CT, and that creates a further independent index that correlated to the patient’s risk of treatment failure and shorter overall survival. It seems likely that the intratumoral structural heterogeneity was critical in training the network, and for this purpose, PET/CT was superior compared with PET/MRI in this study cohort.

Besides tremendous treatment success in advanced melanoma patients, the rapid development of oncologic treatment options comes with increasingly high costs [54] and can cause severe life-threatening side effects [55]. For this purpose, predictive baseline biomarkers are becoming increasingly important for risk stratification and personalized treatment planning. In addition to the histopathological characterization, tumor stage and blood biomarkers, imaging has the potential to play a significant role by extracting and combining the full range of available data. We are at the beginning of implementing AI in oncologic imaging, and it is unclear which data for which tumor type is trend setting and which is redundant. In our prospective study, we performed whole-body examinations of patients before the start of a new systemic treatment (mostly immunotherapy), and we acquired all of the state-of-the-art image data on the same day: contrast-enhanced CT and MRI, as well as the quantitative functional parameters 18F-FDG-PET (glucose consumption) and DWI (diffusivity). Although this may not be a viable approach for everyday clinical practice, it allowed for extensive analysis. In the first step, we investigated more conventional image biomarkers that might be easy to assess in daily routine. Pretherapeutic TMTV is known as a predictive marker for the OS in various malignancies [56], and has also been demonstrated for malignant melanoma undergoing CIT by Son et al. [57], Pires da Silva et al. [58], Ito et al. [59] and Hlongwa et al. [60]. Pires da Silva also describes that patients with smaller total numbers of lesions more frequently had a complete response in his study [58]. We found that cut-off values for the TMTV of 50 mL and four affected organ sites (as simple estimations of the tumor burden of a patient) enabled a good differentiation of patients with longer and shorter OS in our cohort. Nonetheless, there are several patients with a TMTV > 50 or >4 affected organ sites who showed a long OS (e.g., Pat. 13: TMTV: 1982 mL, OS: 1737 days; Pat. 28: five affected organ regions, OS: 869 days). Therefore, a high tumor burden in the baseline scan is not necessarily an expression of high tumor aggressiveness in general or a poor prognosis for an individual patient. When focusing on individual organs, patients with the metastatic involvement of the brain, pleura or spleen did not survive longer than 1108, 210 or 330 days, respectively. Although the numbers of patients in the different groups are low, the high level of the statistical significance of these findings indicates that they are potentially important biomarkers for the clinical outcome of metastasized-melanoma patients. Prior findings match well with our data, showing a limited OS for patients with brain [58,61] liver [58,62] and spleen [58] metastases. Moreover, bone metastasis has been shown to correlate with the lower overall response and shorter progression-free survival, but it did not cause a significantly shorter OS in a prior study [58]. Conversely, LN, soft-tissue and intestinal metastases are known to have a good response to CIT and thus do not affect the OS negatively [58]. We found that at least one lesion with a high Dmlesion correlated significantly with the shorter OS of a patient. In prior studies, a size-dependent response to immunotherapies on the individual-metastasis level has already been shown, resulting in higher rates of complete response for smaller lesions [63]. A suggested explanation is the better drug delivery into the tumor for smaller lesions [63]. Lee et al. has also proven that the response in lesions with complete response was durable (only 3% subsequently progressed) [64], and therefore the impact of small lesions on the patient OS should be limited. Moreover, a low SULpeak in one metastasis of a patient correlated with a shorter OS. Melanoma is usually a highly metabolically active tumor, and this might appear to be a contradiction to our finding that a high tumor burden (MTV, affected organ regions) was also associated with a poorer treatment outcome. A possible explanation might be that the presence of such a low metabolic lesion in advanced-melanoma patients might also represent tumor heterogeneity with the metastasis of poor lymphocytic infiltration, which was shown to correlate with a poor response to immune-activating therapy [65]. Further studies with histological correlations are needed to gain deeper insights into the underlying pathology. The ADC is a quantitative MR parameter for the diffusivity, which is influenced by the cell density or the integrity of the cell membranes, and thus stands for an independent tissue property on a molecular scale. High intraindividual ranges of the ADCmean also correlated with significantly shorter OS, and this might be an expression of intertumoral heterogeneity, which has not been reported previously. However, none of the above imaging indices correlated significantly with the response to the followed systemic treatment. The metabolic activity of the spleen and bone marrow in baseline scans was described as potentially predictive for immunotherapy, although the underlying mechanism for this finding has not been understood so far. An SLR > 1.1 was described in prior studies [8] as a poor prognostic marker for the OS, but the frequency of patients fulfilling this requirement seems to be low: Wong et al. described it as 4–10% of all patients, and we found only one patient in our study cohort (~3%). We could not find an effect of the BLR on the OS in our study. However, even though many of these factors are known and described in the literature, there is a great effort to evaluate these factors manually, hampering their use in clinical routine.

For the AI-assisted evaluation, we found an overall high performance for assessing the risk outcomes in target lesions from the initial imaging. Instead of screening all lesions, we were interested in analyzing the performance (in testing) on preselected targets. The target-lesion selection was based on a combination of a large tumor size (Dmlesion) and high SULpeak, automatically determined by a segmentation network. Overall, the proposed network enables a fast outcome prediction within ~8 s from the imaging data of the initial staging. Misclassifications often occurred in smaller lesions, which led us to conclude that the textural composition was too subtle and not well defined to derive a concise decision. Consequently, the lesion diameter has an important role in the AI-assisted accuracy. To try to understand how decision making in a CNN is made, we analyzed the lesion characteristics for the CNN-based risk groups that were also evaluated in the first part of this study, but we could not show a significant difference between the low- and high-risk groups for any of these clinical parameters. This led to the assumption that other factors, such as the textural characteristics or environmental surrounding, led to a decision regarding the risk classification in the AI-assisted evaluation and can, hence, provide complementary information acting as a potential biomarker for the patient’s risk stratification. Therefore, a comparison of the proposed AI-assisted evaluation to the radiomic features [14] is conceivable, but beyond the scope of this study. We found that training on both imaging modalities has benefits over training on the individual ones. However, only marginal improvements were found over operating on PET/CT alone, except for a statistically significantly improved PPV when considering both imaging modalities. We conjecture that the nonquantitative nature of MR in comparison with CT may lead to the observed reduced performance of PET/MR. As most clinical studies will most likely assess the immunotherapy response with a single imaging modality, we found a good performance for the melanoma-risk stratification of operating on PET/CT alone. However, we want to emphasize that these findings do not generalize to other pathologies or body regions, and they demand further investigation of the role of PET/MR imaging.

We acknowledge further limitations of this pilot study. This is a retrospective analysis of prospectively acquired data, with a limited number of patients with excluded extremities undergoing PET/CT and PET/MR on the same day. Therefore, no external validation was possible, and we decided on internal leave-one-out cross-validation to reduce the possibility of overfitting. Due to the small sample size, the risks of false-positive findings, overfitting or underspecification are very high. A validation of the obtained findings in larger cohorts and on whole-body imaging, including extremities, is therefore necessary. However, the analysis of the whole-body PET/CT and PET/MRI examinations of each patient before systemic treatment is unique in the literature, and especially regarding the inclusion of an AI-assisted analysis. Our evaluation is focused on hybrid imaging from the initial staging before systemic treatment; the clinical parameters, as well as further treatments of course, also influence the clinical outcome. The investigation and impact of follow-up imaging studies would be of interest, but this was not the primary target for this study. It must also be remembered that different systemic therapies were applied, which may thus vary in terms of the response pattern and clinical outcome. Nonetheless, our study cohort reflects a setting from clinical routine with heterogeneous patients, and an imaging-based biomarker is supposed to be independent. We hypothesize that heterogeneity might be a possible driver in our analysis; because it is not ethically feasible to acquire biopsies of all the target lesions (or several lesions), we cannot confirm this statement on a histological level.

## 5. Conclusions

In this pilot study, we confirmed that the overall survival in patients with unresectable metastatic melanoma undergoing systemic treatment correlated with some handcrafted parameters: a high tumor load, metastases in certain organ regions, at least one metastasis with a high diameter or poor metabolism as well as a high intraindividual range of diffusivity. However, the identification of high-risk patients (according to shorter OS and treatment failure) was not feasible with the handcrafted parameters.

We therefore proposed a multistream CNN to provide a complementary in-depth analysis of the disease-progression and mortality-risk outcomes using multiparametric imaging data from PET, CT and MRI. Risk stratification was feasible with high specificity and sensitivity. We found no statistically significant correlation to the handcrafted extracted parameters, which indicates that a CNN can provide in-depth risk information that is complementary to the handcrafted parameters. We hypothesized that the lesions’ textural heterogeneity here guided the risk evaluation, and for this purpose, PET/CT was superior to PET/MRI in our cohort.

Due to the small sample size, the efficiency and reliability have to be further confirmed in studies with larger cohorts. Nonetheless, we see a potential role for multiparametric image analysis paired with an AI-assisted evaluation operating on the initial imaging staging. The analysis can serve as an additional method for risk stratification, and it can support personalized treatment decisions for oncology in the future.

## Figures and Tables

**Table 1:** Patient characteristics.

	Sex	Age	Therapeutic Agent	Response ^†^	OS	Risk
1	m	49	PD1-Ab	progress	330 *	High
2	m	52	CTLA4-Ab + BRAF/MEK inhibitor	progress	127 *	High
3	f	59	CTLA4-Ab	progress	495 *	High
4	m	75	PD1-Ab	partial response	1266 *	Low
5	f	74	PD1-Ab	progress	814 *	High
6	f	74	CTLA4-Ab	progress	170 *	High
7	f	53	PD1-Ab	progress	148 *	High
8	m	51	CTLA4-Ab	complete response	1737	Low
9	f	49	PD1-Ab	complete response	1732	Low
10	m	59	CTLA4-Ab	progress	453 *	High
11	f	62	BRAF inhibitor	progress	277 *	High
12	f	52	CTLA4-Ab + V-TEC	progress	1456	High
13	m	84	PD1-Ab	partial response	1512	Low
14	m	35	BRAF/MEK inhibitor	progress	210 *	High
15	f	64	PD1-Ab	progress	202 *	High
16	m	81	BRAF/MEK inhibitor	partial response	869 *	Low
17	f	58	PD1-Ab	progress	1108 *	High
18	f	83	PD1-Ab	progress	448 *	High
19	m	75	PD1-Ab	progress	158 *	High
20	m	66	PD1-Ab	stable disease	906 *	Low
21	m	76	BRAF/MEK inhibitor	partial response	243 *	High
22	m	73	PD1-Ab	partial response	1302	Low
23	m	64	PD1-Ab	progress	1240	High
24	m	60	PD1-Ab	complete response	1172	Low
25	f	67	PD1-Ab + CTLA4-Ab	partial response	1222	Low
26	f	68	PD1-Ab + CTLA4-Ab	partial response	1157	Low
27	f	50	BRAF/MEK inhibitor	complete response	362 *	High
28	m	56	PD1-Ab	progress (iRECIST, stable disease)	1101	High
29	f	40	PD1-Ab	stable disease	1020	Low
30	f	73	PD1-Ab	progress	1021	High
31	f	82	PD1-Ab + CTLA4-Ab	progress	976	High
32	f	53	PD1-Ab + CTLA4-Ab	partial response	847	Low
33	m	44	PD1-Ab	progress	834	High
34	m	40	PD1-AK + CDK4/6	progress	123 *	High
35	f	57	PD1-Ab + CTLA4-Ab	progress	174 *	High
36	f	73	PD1-Ab	progress	675	High
37	m	61	PD1-Ab + CTLA4-Ab	progress	120 *	High

^†^: Response was evaluated 3 months after the start of therapy by whole-body 18F-FDG PET/CT (or in cases of obvious clinical progress, earlier by other cross-section imaging); *: patient died; OS: overall survival in days; m: male; f: female; Ab: antibody; PD1: programmed cell death protein 1; MEK: mitogen-activated protein kinase kinase; CTLA4: cytotoxic T-lymphocyte-associated protein 4; CDK4/6: cyclin-dependent kinases 4/6.

**Table 2:** Results of lesion evaluation in patient cohort.

	Mean	Range
Number of lesions per patient	26	2–200+
Total metabolic tumor volume (mL)	106	1–1982
Total lesion glycolysis	560	1–13,341
Average lesion size per patient (mm)	16	5–48
Intraindividual range in lesion size (mm)	41	4–295
Average ADC_mean_ per patient (mm^2^/s)	1039	459–1782
Intraindividual range in ADC_mean_ (mm^2^/s)	599	4–1513
Average SUL_peak_ per patient	3.4	0.6–11.2
Intraindividual range in SUL_peak_	4.0	0.0–13.9
Number of affected organ regions	3	1–8
**Number of Patients with Metastases in Certain Organ Regions**		
Lymph nodes		17
Soft tissue		18
Bone		13
Liver		15
Spleen		5
Lung		17
Pleura		3
Brain		10
Other viscera (excluding liver and spleen)		9

**SULpeak**: peak standardized uptake value corrected by lean body mass; **ADC**: apparent diffusion coefficient.

## Data Availability

Not applicable.

## Supplementary Materials

The following supporting information can be downloaded at: https://www.mdpi.com/article/10.3390/diagnostics12092102/s1, Supporting Figure S1: Kaplan–Meier plot for overall-survival analysis since start of systemic treatment for all patients depending on total lesion glycolysis (TLG) and total number of lesions.
