# Supplementary figures and images for: Development of a Hybrid-Imaging-Based Prognostic Index for Metastasized-Melanoma Patients in Whole-Body 18F-FDG PET/CT and PET/MRI Data
Source: Diagnostics (Basel). 2022 Aug 30;12(9):2102. doi: 10.3390/diagnostics12092102 (PMC9498091; doi:10.3390/diagnostics12092102)

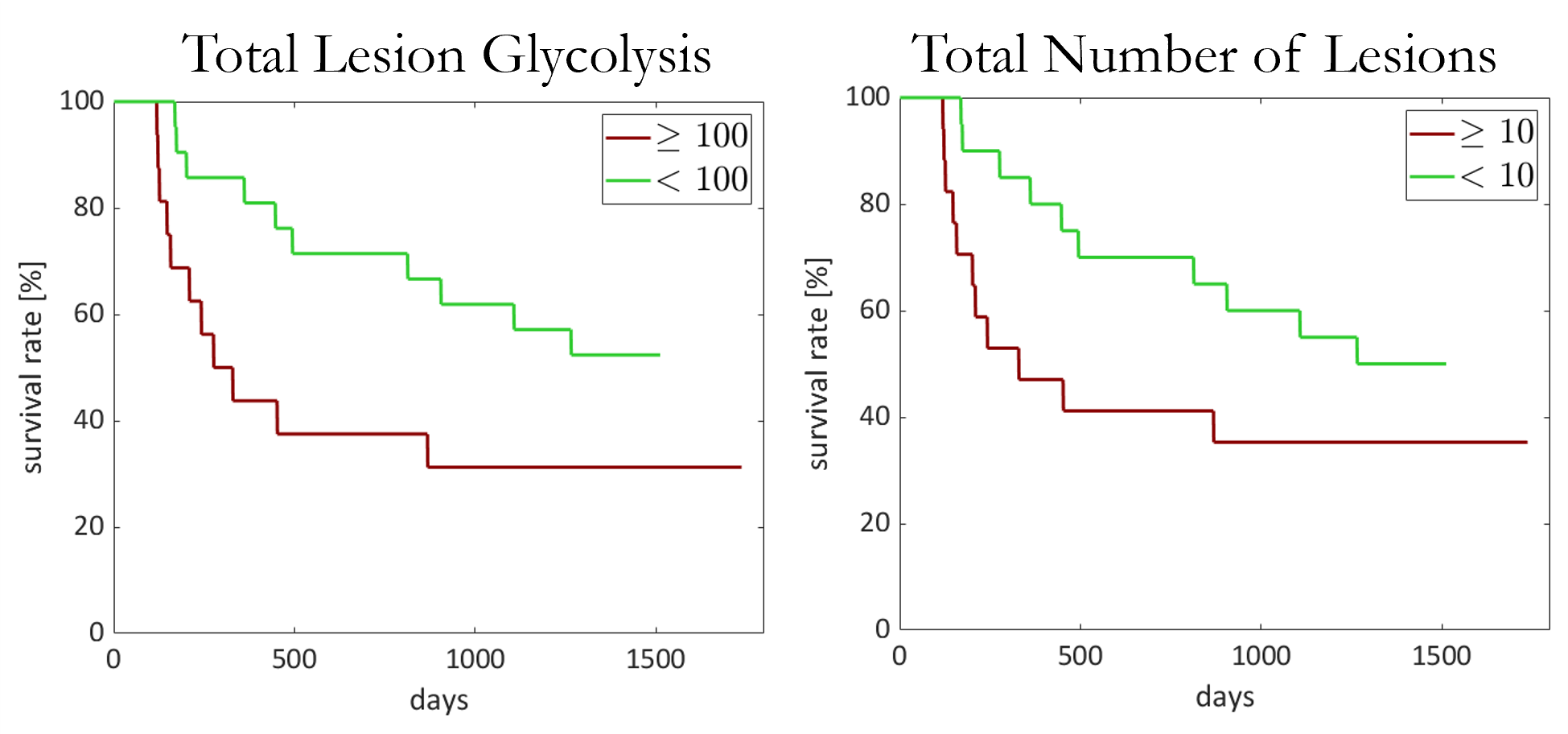

Supplement: Supplementary file 1 [file diagnostics-12-02102-s001.zip › Suppl Figure S1.tif]
